# Impact of physical activity on the association between lipid profiles and mortality among older people

**DOI:** 10.1038/s41598-017-07857-7

**Published:** 2017-08-21

**Authors:** Shuo-Ming Ou, Yung-Tai Chen, Chia-Jen Shih, Der-Cherng Tarng

**Affiliations:** 1 Division of Nephrology, Department of Medicine, Taipei Veterans General Hospital, Taipei, Taiwan; 2 School of Medicine, National Yang-Ming University, Taipei, Taiwan; 3 Institute of Clinical Medicine, National Yang-Ming University, Taipei, Taiwan; 4 Department of Medicine, Taipei City Hospital Heping Fuyou Branch, Taipei, Taiwan; 5 Department of Medicine, Taipei Veterans General Hospital, Yuanshan Branch, Yilan, Taiwan; 6 Department and Institute of Physiology, National Yang-Ming University, Taipei, Taiwan

## Abstract

High serum lipid levels are independent predictors of mortality risk in the general population. Recent data suggest that this may not apply in the older populations, and even acts in the opposite direction. In consideration of the frail state, minimum amount of physical activity (60–100 minutes each week) may be more suitable for older individuals but its role in lipid profiles has never been explored. Between 2006 and 2010, we conducted a cohort study of 83,820 participants aged ≥65 years using the Taipei City Elderly Health Examination Database. Participants were classified as inactive, low or high in their level of physical activity. Older individuals with lowest quintile of total cholesterol, non-HDL and HDL were associated with increased risk of all-cause mortality compared to those with other quintile of these lipid profiles. Compared to inactive older individuals, both low (adjusted hazard ratios [aHR] 0.75, 95% confidence interval [CI] 0.70–0.81) and high active older individuals (aHR 0.55, 95% CI 0.51–0.59) were associated with lower risks of mortality. Physical activity, even minimum volume of exercise, in older people has to be encouraged to reduce the increased risk of mortality from low serum lipid levels.

## Introduction

The world’s populations are on a remarkable transition to “aged society”, with estimated numbers of individuals over the age of 65 increasing from 7.9% by 2010 to 17% by 2050^1, 2^. The burden of cardiovascular disease has been increasing after 65 years, and these people have multiple co-morbidities, which poses major challenges for health care clinicians to management of modifiable cardiovascular risk factors. The Adult Treatment Panel -III guidelines recommended that optimal low-density lipoprotein (LDL) level is <100 mg/dL, but an LDL-C goal of <70 mg/dL is a therapeutic option in high-risk patients^3, 4^. However, the older population requires special consideration because many risk reduction strategies applied in the general population may not as reliable as desired in this older population (sometimes even in the opposite direction). Indeed, there are issues and some controversy concerning the high serum lipid level as a risk factor for mortality among the older people.

Some but not all studies have suggested that increased serum lipids and lipoproteins were associated with increased risks of all-cause or cardiovascular mortality in older patients^5–7^, whereas other studies have found no such correlation^8^, or even an inverse correlation^9–11^. However, previous studies were limited by their relatively small sample size, short follow-up period, single hospital data and lack consideration of effect of exercise on serum lipids. A meta-analysis of 1,166 participants aged over 60 years found exercise enables to improve anthropometric measures (i.e., body mass index and waist circumference), but this meta-analysis suggested the effect of exercise on serum lipids remains unclear^12^.

Physical activity of older people can improve quality of life, musculoskeletal and psychological health as well as lead to improving cardiovascular risks and mortality in older people^13, 14^. In general, current Centers for Disease Control and Prevention suggested 150 min or more per week of moderate intensity aerobic activity met the exercise recommendation in adults^15^. However, previous study found that minimum amount of physical activity (60–100 minutes or more per week of moderate-intensity exercise) also exert survival benefits, even in individuals at risk of cardiovascular disease or older individuals with heart failure^16, 17^. In consideration of the frail state of older individuals, minimum amount of physical activity may be more suitable for this special population. Thus, the aim of study was to assess whether minimum amount of physical activity could reduce the risk of mortality from lipid disorder among individuals aged ≥65 years over a five-year follow-up period. To address this knowledge gap in the present study is particularly important to validate that older people with consistent and proper exercise could live longer and healthy.

## Methods

### Study Design and Population

This study was a large-scaled community-dwelling cohort analysis based on the records of the Taipei City Elderly Health Examination Database, which was designed to provide annual health examinations for citizens aged ≥65 years reimbursed by Taipei City Government^18^. All Taipei citizens who met age criteria were encouraged to participate in this voluntary health examination program and signed informed consent to use their medical records for the research purpose. In addition, Taipei City Government has contracted with the hospitals to ensure that all qualified hospitals have used identical standardized examination protocol. The detailed information on the health examination and data offered by Taipei City Elderly Health Examination Database has been described previously^19, 20^. In addition, the mortality information on these participants were collected by linking our database with the Taiwan’s National Death Registry through a unique personal identification number.

As every elderly participant was enrolled, his or her informed consent is obtained to authorize the Taipei City Government Institution to process health examination data for the research purpose. Detailed information on the health examination and data is stored centrally in the Taipei City Elderly Health Examination Database and is de-identified before releasing it to protect the privacy. This study was approved by the institutional review board of Taipei City Hospital (TCHIRB-1010323-E; TCHIRB-1030601-W). In our study, all methods were performed in accordance with relevant guidelines and regulations.

### Data collection

From Taipei City Elderly Health Examination Database, we extracted the data from older participants who had serum total cholesterol, TG, and HDL measured at least once between 2006 and 2010. The participants who had no record of daily physical activity or had history of end-stage renal disease were excluded from the analysis. Data on socio-demographic and lifestyle-related variables (i.e., age, gender, smoking, and alcohol consumption), exercise habits as well as medical and drug history were collected by specially designed questionnaires of the Geriatric Health Examination Program. The duration and intensity of weekly physical activity were converted into MET-hour/week, which was based on Ainsworth’s compendium of physical activities. Physical activities were classified as ‘inactive’, ‘low active’ and ‘high active’ and defined as follow: high active met the current exercise recommendation of 60–100 minutes per week, ‘low active’ exercise less than 60 minutes per week, and ‘inactive’ without exercise. At each visit, anthropometric parameters, including body height, body weight and blood pressure were measured and recorded. BMI was calculated as body weight (kg) divided by body height (m) squared. Blood samples were collected after overnight fast for measurement of complete blood cell count; blood urea nitrogen, serum creatinine, albumin, uric acid, and fasting glucose levels. Non-HDL level was calculated as total cholesterol minus HDL. The estimated glomerular filtration rate (eGFR) was calculated by the CKD-EPI equation Missing data were replaced by mean imputation in the entire cohort.

### Outcomes

The Mortality data were obtained from Taiwan’s National Death Registry and death certificates were coded in accordance with the International Classification of Diseases (ICD)-9 or ICD-10. The outcome of interest was all-cause mortality (ICD-9 001.x–999.x or ICD-10 A00.x–Z99.x). In this study, all participants were followed until death or 31 December 2010.

### Statistical Analysis

All participants were divided into three groups according to their physical activity status. Differences in the distributions of continuous and ordinal variables were tested using the Kruskal-Wallis test, and categorical variable were tested using the chi-square test. Serum lipid and lipoprotein levels were divided into quintiles according to the distribution in the participants. The Kaplan-Meier method was used to estimate the cumulative incidence of death in our study cohorts, and log-rank test was used to assess differences between groups. Cox proportional hazard models were used to calculate the hazard ratios (HRs) of mortality and their 95% confidence intervals (CIs). The adjusted HR was analyzed after adjusting for age, sex, body mass index, smoking, alcohol use, systolic blood pressure, diastolic blood pressure, hypertension, diabetes mellitus, coronary artery disease, cerebrovascular disease, white blood cell count, hemoglobin, albumin, geriatric nutritional risk index, uric acid, fasting glucose, estimated glomerular filtration rate, and urine protein level. Interaction between physical activity and each serum lipid and lipoprotein level on mortality was also tested by likelihood ratio test. When an interaction test is significant, subgroup analysis is performed accordingly. All statistical analyses were conducted using STATA statistical software (version 13.0; StataCorp, College Station, Texas, USA). Statistical significance was defined as *P* < 0.05.

## Results

### Clinical Characteristics of the Study Population

A total of 83,820 older individuals enrolled in this study, with a mean age of 73.6 ± 6.7 years and a mean BMI of 24.2 ± 3.5 kg/m^2^. This study cohort was slightly female-predominant (51.7%) with the prevalence of comorbidities as follows: hypertension, 41.7%; diabetes mellitus, 13.0%; coronary artery disease, 17.7%; and cerebrovascular disease, 0.9%. Table 1 shows the comparison of the demographic characteristics, comorbid diseases, lipid profiles and other laboratory values between inactive, low active and high active older individuals.

**Table 1:** Demographic and Clinical Characteristics of the Study Population.

Characteristic	Overall	Physical Activity Status			*P*
		Inactive	Low Active	High Active	
Number of patients	83,820	10,474	28,151	45,195	
Male	40,520 (48.3)	4,086 (39.0)	12,461 (44.3)	23,973 (53.0)	<0.001
Demographic characteristics					
Age, years	73.6 (6.7)	75.4 (7.5)	73.5 (6.8)	73.2 (6.4)	<0.001
BMI, kg/m^2^	24.2 (3.5)	24.3 (4.0)	24.3 (3.6)	24.1 (3.3)	<0.001
Smoking	6,603 (7.9)	1,087 (10.4)	2,419 (8.6)	3,097 (6.9)	<0.001
Alcohol use	15,260 (18.2)	1,240 (11.8)	4,718 (16.8)	9,302 (20.6)	<0.001
Systolic blood pressure, mm Hg	135.0 (19.8)	135.4 (20.9)	134.9 (19.9)	134.9 (19.5)	0.068
Diastolic blood pressure, mm Hg	75.6 (11.9)	75.5 (12.4)	75.7 (11.8)	75.5 (11.8)	0.288
Comorbid disease, n (%)					
Hypertension	34,961 (41.7)	4,506 (43.0)	11,739 (41.7)	18,716 (41.4)	0.011
Diabetes mellitus	10,901 (13.0)	1,656 (15.8)	3,683 (13.1)	5,562 (12.3)	<0.001
Coronary artery disease	14,821 (17.7)	2,110 (20.1)	5,032 (17.9)	7,679 (17.0)	<0.001
Cerebrovascular disease	784 (0.9)	207 (2.0)	279 (1.0)	298 (0.7)	<0.001
Lipid profile, mean (SD)					
Total cholesterol, mg/dL	197.6 (35.9)	197.8 (38.6)	198.6 (36.3)	196.9 (35.0)	<0.001
Non-HDL, mg/dL	143.8 (34.6)	145.1 (37.2)	145.5 (35.0)	142.4 (33.7)	<0.001
HDL, mg/dL	53.4 (14.7)	52.7 (14.8)	53.1 (14.5)	54.5 (14.8)	<0.001
Triglyceride, mg/dL	122.6 (78.7)	131.5 (84.5)	127.4 (84.0)	117.6 (73.2)	<0.001
Other laboratory data, mean (SD)					
WBC count,/mm^3^	6016 (1879)	6366 (2270)	6093 (1982)	5888 (1690)	<0.001
Hemoglobin, g/dL	13.5 (1.4)	13.2 (1.6)	13.4 (1.4)	13.6 (1.4)	<0.001
Albumin, g/dL	4.3 (0.3)	4.2 (0.4)	4.3 (0.3)	4.4 (0.3)	<0.001
GNRI	113.2 (10.0)	113.0 (11.6)	113.5 (10.0)	113.0 (9.6)	<0.001
Uric acid, mg/dL	5.9 (1.6)	5.9 (1.8)	5.9 (1.6)	5.9 (1.5)	0.649
Fasting glucose, mg/dL	106.1 (27.8)	107.7 (34.0)	106.1 (28.7)	105.6 (25.5)	<0.001
eGFR, mL/min/1.73 m^2^	69.0 (17.5)	65.6 (19.2)	68.6 (17.9)	69.9 (16.7)	<0.001
Urine protein level, n (%)					<0.001
Negative	71,894 (85.8)	8,330 (79.5)	23,913 (84.9)	39,651 (87.7)	
Trace	4,991 (6.0)	834 (8.0)	1,790 (6.4)	2,367 (5.2)	
+	4,264 (5.1)	709 (6.8)	1,509 (5.4)	2,046 (4.5)	
+ + and more	2,351 (2.8)	492 (4.7)	832 (3.0)	1,027 (2.3)	

Data are presented as *n* (%) or mean ± standard deviation.

Abbreviations: BMI, body mass index; SD, standard deviation; HDL, high-density lipoprotein; WBC, white blood cell; GNRI, Geriatric nutritional risk index; eGFR, estimated glomerular filtration rate.

### The Association between Lipid Level, Physical Activity Status, and Future Risks of Mortality among Older People

**Figure 1:**
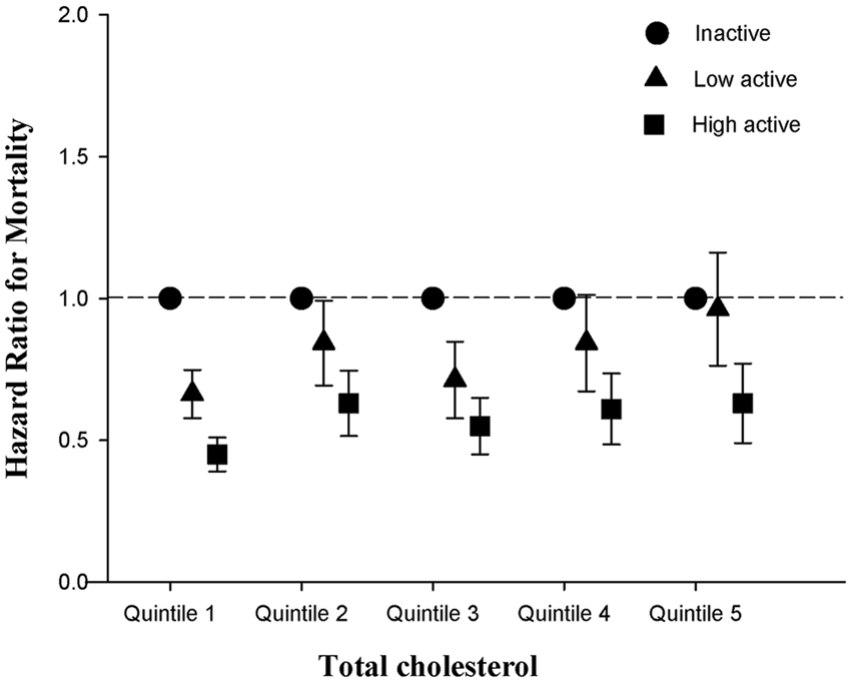
Adjusted hazard ratios for mortality based on the quintiles of total cholesterol and physical activity status among older individuals.

**Figure 2:**
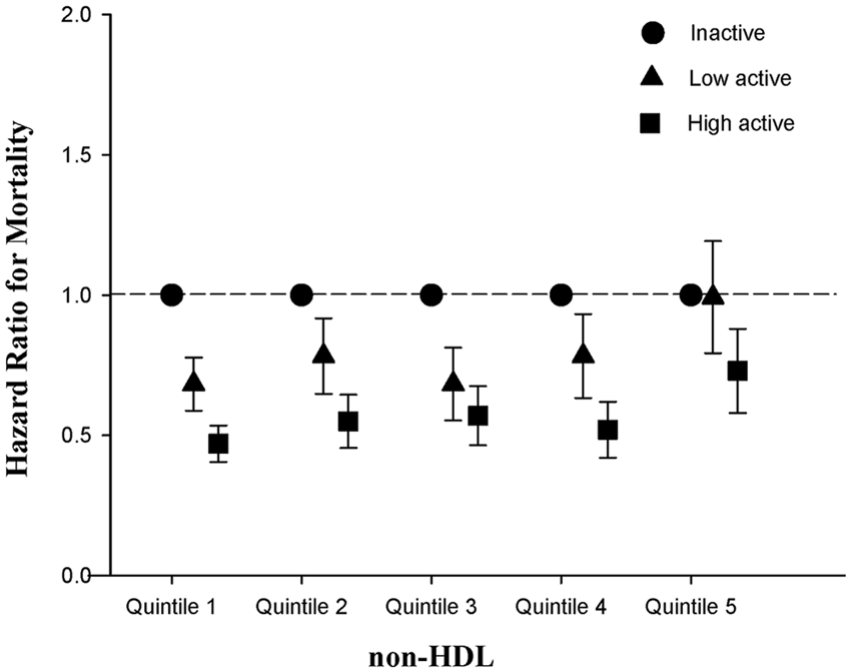
Adjusted hazard ratios for mortality based on the quintiles of non-high-density lipoprotein cholesterol and physical activity status among older individuals.

Baseline distributions of each lipid variable as well as the future mortality events are provided in Table 2. During the mean follow-up period of 3.6 years, there were 5,040 deaths (6.0%) with all-cause mortality rate of 17.9 per 1,000 person-years. In multivariable Cox regression models, older individuals with lowest quintile of total cholesterol, non-HDL and HDL were associated with increased risk of all-cause mortality compared to those with other quintile of these lipid profiles (Table 2, Figs 1 and 2). Of note, the association of low triglyceride levels and increased risk of all-cause mortality became insignificant after adjusting for other confounding variables (Table 2). As shown in Table 3, both low (aHR 0.75, 95% confidence interval [CI] 0.70–0.81) and high active older individuals (aHR 0.55, 95% confidence interval [CI] 0.51–0.59) were associated with lower risks of mortality compared to inactive individuals (*P* for trend < 0.001).

**Table 2:** Mortality Risks among Older Participants According to Baseline Lipid Levels.

Characteristic	Quintile 1	Quintile 2	Quintile 3	Quintile 4	Quintile 5
Total Cholesterol					
Median (range), mg/dL	154 (<169)	179 (169–187)	196 (188–205)	215 (206–227)	244 (>227)
No. of events	1,676	1,022	887	786	696
HR (95% CI)	1	0.64 (0.59–0.69)	0.54 (0.50–0.59)	0.48 (0.44–0.53)	0.45 (0.41–0.49)
P value		<0.001	<0.001	<0.001	<0.001
aHR (95% CI) ^*^	1	0.84 (0.77–0.91)	0.82 (0.75–0.89)	0.85 (0.77–0.93)	0.83 (0.75–0.91)
P value		<0.001	<0.001	<0.001	<0.001
Non-HDL					
Median (range), mg/dL	102 (<116)	126 (116–134)	143 (135–151)	160 (152–171)	188 (>171)
No. of events	1,520	1,080	871	791	805
HR (95% CI)	1	0.71 (0.66–0.77)	0.59 (0.54–0.64)	0.56 (0.52–0.61)	0.55 (0.51–0.60)
P value		<0.001	<0.001	<0.001	<0.001
aHR (95% CI) ^*^	1	0.90 (0.83–0.98	0.87 (0.80–0.95)	0.91 (0.83–0.99)	0.91 (0.82–0.99)
P value		0.014	0.002	0.035	0.028
HDL					
Median (range), mg/dL	39 (<43)	46 (43–48)	50 (49–52)	60 (53–66)	75 (>66)
No. of events	1,665	1,050	738	888	726
HR (95% CI)	1	0.60 (0.56–0.65)	0.61 (0.47–0.56)	0.61 (0.56–0.66)	0.48 (0.44–0.53)
P value		<0.001	<0.001	<0.001	<0.001
aHR (95% CI) ^*^	1	0.75 (0.70–0.82)	0.73 (0.66–0.80)	0.87 (0.79–0.95)	0.72 (0.65–0.79)
P value		<0.001	<0.001	0.002	<0.001
Triglyceride					
Median (range), mg/dL	57 (<70)	81 (70–92)	105 (93–119)	138 (120–162)	206 (>162)
No. of events	1,229	1,083	968	904	883
HR (95% CI)	1	0.90 (0.82–0.97)	0.80 (0.73–0.87)	0.76 (0.69–0.82)	0.75 (0.69–0.82)
P value		0.008	<0.001	<0.001	<0.001
aHR (95% CI)^*^	1	1.04 (0.96–1.14	1.03 (0.95–1.13)	1.05 (0.95–1.15)	1.08 (0.98–1.19)
P value		0.329	0.457	0.352	0.139

^*^Adjusted for age, sex, body mass index, smoking, alcohol use, systolic blood pressure, diastolic blood pressure, hypertension, diabetes mellitus, coronary artery disease, cerebrovascular disease, white blood cell count, hemoglobin, albumin, geriatric nutritional risk index, uric acid, fasting glucose, estimated glomerular filtration rate, and urine protein level.

Abbreviations: HR, Hazard ratio; aHR, adjusted hazard ratio; CI, confidence interval; HDL, high-density lipoprotein.

**Table 3:** Association Between Physical Activity Status and Mortality in Older Participants.

Physical activity status	No. of Subjects	No. of Event	Person Years	Incidence Rate^*^	Cox Regression Analysis			
					Crude HR (95% CI)	*P*	Adjusted^†^ HR (95% CI)	*P*
Inactive	10,474	1,219	32,248	37.80	1		1	
Low active	28,151	1,887	93,796	20.12	0.53 (0.49–0.56)	<0.001	0.75 (0.70–0.81)	<0.001
High active	45,195	1,934	155,634	12.43	0.32 (0.30–0.35)	<0.001	0.55 (0.51–0.59)	<0.001

^*^Per 10^3^ person years.

^†^Adjusted for age, sex, body mass index, smoking, alcohol use, systolic blood pressure, diastolic blood pressure, hypertension, diabetes mellitus, coronary artery disease, cerebrovascular disease, white blood cell count, hemoglobin, albumin, geriatric nutritional risk index, uric acid, fasting glucose, estimated glomerular filtration rate, and urine protein level.

Abbreviations: HR, Hazard ratio; CI, confidence interval; HDL, high-density lipoprotein.

### The Interaction between Lipid Level and Physical Activity Status on Future Risks of Mortality among Older People

**Figure 3:**
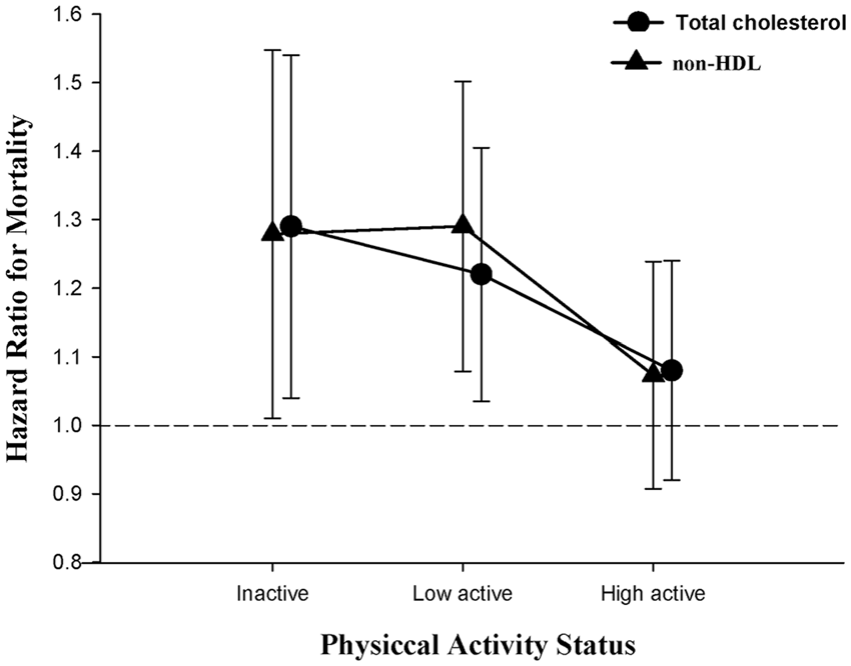
Mortality risks by physical activity status among individuals in the highest quintile of total cholesterol or non-high-density lipoprotein cholesterol.

Test of interaction was performed and significant for physical activity status and non-HDL (*P*_interaction_ = 0.01; Table 4), whereas interaction did not reach statistical significance for physical activity status and total cholesterol (*P*_interaction_ = 0.06; Table 4), HDL (*P*_interaction_ = 0.295, Appendix Table 1) or triglyceride (*P*_interaction_ = 0.124; Appendix Table 2). When stratified by physical activity status, both lower total cholesterol and lower non-HDL (Fig. 3; Appendix Tables 3 and 4) increased risks of mortality in inactive and low active older individuals. However, these associations disappeared in high active older individuals. When stratified by lipid profiles, high active older individuals were significantly associated with increased long-term survival in all subgroups (Appendix Table 5).

**Table 4:** Mortality Risks by Total Cholesterol Levels and Non-HDL Levels, and By Physical Activity Status Among Older Participants.

	Inactive			Low active			High active		
	Event (%)	Crude HR (95% CI)	Adjusted HR^*^ (95% CI)	Event (%)	Crude HR (95% CI)	Adjusted HR^*^ (95% CI)	Event (%)	Crude HR (95% CI)	Adjusted HR^*^ (95% CI)
Total Cholesterol^†^									
Quintile 1	486 (20.8)	1	1	612 (10.8)	1	1	578 (6.1)	1	1
Quintile 2	216 (11.3)	0.50 (0.42–0.59)	0.65 (0.55–0.78)	379 (7.0)	0.64 (0.56–0.73)	0.85 (0.74–0.97)	427 (4.7)	0.77 (0.68–0.88)	0.97 (0.86–1.11)
Quintile 3	205 (10.1)	0.46 (0.39–0.54)	0.75 (0.63–0.90)	315 (5.6)	0.50 (0.44–0.58)	0.79 (0.68–0.91)	367 (4.0)	0.65 (0.57–0.74)	0.91 (0.79–1.04)
Quintile 4	166 (8.2)	0.36 (0.30–0.43)	0.72 (0.60–0.88)	303 (5.3)	0.48 (0.42–0.55)	0.85 (0.73–0.99)	317 (3.5)	0.58 (0.51–0.67)	0.93 (0.80–1.07)
Quintile 5	159 (7.3)	0.32 (0.27–0.38)	0.63 (0.51–0.77)	285 (5.0)	0.46 (0.40–0.53)	0.89 (0.76–1.04)	252 (3.0)	0.52 (0.45–0.60)	0.90 (0.77–1.06)
Non-HDL^‡^									
Quintile 1	424 (19.0)	1	1	541 (9.9)		1	555 (5.8)	1	1
Quintile 2	244 (12.1)	0.60 (0.51–0.70)	0.82 (0.69–0.98)	406 (7.3)	0.73 (0.64–0.83)	0.92 (0.81–1.06)	430 (4.5)	0.78 (0.69–0.89)	0.94 (0.83–1.08)
Quintile 3	201 (10.4)	0.51 (0.43–0.60)	0.83 (0.69–0.997)	307 (5.5)	0.54 (0.47–0.62)	0.81 (0.70–0.94)	363 (4.0)	0.70 (0.61–0.80)	0.96 (0.83–1.10)
Quintile 4	192 (9.8)	0.47 (0.40–0.56)	0.88 (0.73–1.06)	310 (5.7)	0.55 (0.48–0.64)	0.94 (0.81–1.09)	289 (3.4)	0.61 (0.53–0.70)	0.89 (0.77–1.03)
Quintile 5	171 (7.3)	0.35 (0.30–0.42)	0.67 (0.54–0.82)	330 (5.4)	0.54 (0.47–0.62)	0.95 (0.82–1.10)	304 (3.6)	0.66 (0.57–0.76)	1.00 (0.86–1.16)

^*^Adjusted for age, sex, body mass index, smoking, alcohol use, systolic blood pressure, diastolic blood pressure, hypertension, diabetes mellitus, coronary artery disease, cerebrovascular disease, white blood cell count, hemoglobin, albumin, geriatric nutritional risk index, uric acid, fasting glucose, estimated glomerular filtration rate, and urine protein level.

^†^Interaction for total cholesterol and physical activity status, p value = 0.06.

^‡^Interaction for non-HDL and physical activity status, p value = 0.01.

Abbreviations: HR, Hazard ratio; CI, confidence interval.

## Discussion

To our best knowledge, to date, this is the largest older specific study to assess the relationship between serum lipid profiles and long-term mortality outcome, as well as the first cohort study to evaluate the modifying effect of physical activity in its association. Our results have confirmed that low total cholesterol or low non-HDL-C levels were associated with increased risk of overall mortality. In addition, we found that the associations became disappear after considering older participants who performed minimum amount of physical activity.

There is conflicting evidence with regard to the cardiovascular risk associated with total cholesterol or LDL-C in older populations as some data suggested an increased risk association^5–7^, whereas others favor inverse pattern associations^9–11^. To address this issue, one study calculated pooled risk estimates from 22 international cohort studies suggested elevated cholesterol level are associated with increased (but relative lower) risk of coronary disease in older people^21^. However, the pooled results should be interpreted cautiously since the evidences were gathered from the analysis of mixed population (age 43–75 years). It is still needed more refined and uniform studies with extended follow-up periods and adjusting for other covariates. In the present study, we observed that even using two different lipoproteins (total cholesterol and non-HDL), there was an inverse association with all-cause mortality. This corroborates the results of the randomized control trial (the European Working Party on High Blood Pressure in the Elderly study) consisting of 822 patients aged >60 years which found that serum total cholesterol was independently and inversely correlated with mortality. However, the EWPHBPE study included 70% women subjects which then might thus limit the broadness of its clinical application^22^.

Previous studies suggested that high TC and non-HDL levels among older populations might be associated with higher BMI and better general health conditions. In contrast, low TC and non-HDL levels may be related to nutritional deficiencies and poor health conditions in fragility populations, such as dialysis and older patients, which may explain the reverse epidemiology regard to cholesterol/non-HDL inversely associated with mortality. However, Cabrera *et al*. found that higher mortality were still found among older individuals with low total cholesterol even after exclusion of those whose BMI <20.0 kg/m^2^ or died in the first two years^23^. In addition, Tuikkala *et al*.^24^ conducted another study of a community-dwelling older population aged 75 years and older and found the association between low total cholesterol levels and mortality was independent of underlying comorbidities and general health condition. Therefore, the reason why low serum lipid levels represent poor prognostic indicators remained unclear, which may be independent of BMI, underlying comorbidities and even general health conditions. In the present study, we found that physical activity may confound the association between low TC/non-HDL and mortality. The risk of mortality was not increased in older individuals with regular physical activity, whereas in those without physical activity, the risk of mortality increased in low TC/non-HDL groups.

As people get older, low cholesterol levels may not only reflect nutritional intake but also seems to be a surrogate for frailty and disability resulted from chronic illness and inflammatory status^11, 25–27^. Older individuals are most likely to be inactive because of comorbidities, loss of balance and diminished functional capacity^28–30^. In contrast, physical activity has favorable effects in lowering lipids and lipoproteins levels^31, 32^. Previous studies found that physical activity may be of particular value in generating anti-inflammatory response and anti-oxidant effects^33, 34^. Physical activity is also associated with reducing atherogenic lipid profiles^35^, lowering blood pressure, improving insulin resistance and improving endothelial dysfunction, which may therefore showing a better prognosis in older individuals with lower cholesterol levels (e.g., healthy low cholesterol levels). In addition, increasing physical activity is found to help improve quality of life in the older individuals^36^. A cross-sectional study consisting of 321 community dwelling older people found physical activity indirectly influencing quality of life from baseline to 18 months via increasing self-worth and reducing disability limitations independent of baseline demographic conditions^37^. Another Australian longitudinal study showed that physical activity was associated with improving quality of life even in older women with the history of depressive symptoms. Therefore, increasing physical activity may be associated with improvement for both physical and mental health-specific quality of life in the older individuals^38^.

The availability of information on physical activity and mortality for a total of 83,820 older people over an extended follow-up period is the main strength of our study. To address these points, the present study enrolled older individuals attending a geriatric health examination and they are relatively healthy. Therefore, it is less likely that profound malnutrition, severe illness and/or bed-ridden status would confound our results. However, our study still has some limitations. First, as the nature of the Elderly Health Examination Database, we only focused on the “risk assessment strategy” for older individual explore the association of lipid disorders with all-cause mortality. However, we did not investigate the effects of lipid-lowering treatments; therefore, with regard to therapeutic intervention with lipid-lower drugs in terms of “risk reduction strategy” may require other further clinical studies conducted in the older population. Second, serum LDL-C levels in our dataset were not measured directly but were calculated according to Friedewald’s equation. Therefore, we used non-HDL instead of LDL-C as the principal marker of pro-atherogenic lipoproteins in order to avoid the bias for the estimation of LDL-C levels in Friedewald’s equation^39–41^. Previous studies have found non-HDL levels were the best predictors for coronary artery events among all cholesterol measures^40, 42^. Finally, the physical activity measure in the present study is self-reported and therefore this could lead to recall bias and over-report physical activity because of social desirability biases. However, because over-report physical activity may lead misclassification (i.e., non-physically active groups into physically active groups), such misclassification would most likely have underestimated the risk reduction effects of physical activity on mortality. In addition, the Centers for Disease Control and Prevention released guideline to provide information and guidance on the amount of physical activity based on previous epidemiologic studies of the association between self-reported physical activity and potential benefits.

In conclusion, our study demonstrated that the association between low serum cholesterol/non-HDL and increased risks of mortality in the older population and this association disappeared when considering older people who participate in regular physical activity. Confronting the low serum cholesterol/non-HDL levels in older people or patients, we recommend that physicians should be alert to find out more information on the connection between low cholesterol and health behaviors such as physical activity.

## Electronic supplementary material

Supplementary Information
