# Supplementary material for: Impact of physical activity on the association between lipid profiles and mortality among older people
Source: Sci Rep. 2017 Aug 21;7:8399. doi: 10.1038/s41598-017-07857-7 (PMC5567179; doi:10.1038/s41598-017-07857-7)
Supplement: Supplementary file 1 — Supplementary Information [file 41598_2017_7857_MOESM1_ESM.doc]

**Impact of physical activity on the association between lipid profiles and mortality among older people**

Shuo-Ming Ou, MD*^1,2,3^, Yung-Tai Chen, MD*^2,4^, Chia-Jen Shih, MD^2,5^, Der-Cherng Tarng, MD, PhD^1,2,3,6^

1. Division of Nephrology, Department of Medicine, Taipei Veterans General Hospital, Taipei, Taiwan
2. School of Medicine, National Yang-Ming University, Taipei, Taiwan
3. Institute of Clinical Medicine, National Yang-Ming University, Taipei, Taiwan
4. Department of Medicine, Taipei City Hospital Heping Fuyou Branch, Taipei, Taiwan
5. Department of Medicine, Taipei Veterans General Hospital, Yuanshan Branch, Yilan, Taiwan
6. Department and Institute of Physiology, National Yang-Ming University, Taipei, Taiwan

* Shuo-Ming Ou and Yung-Tai Chen contributed equally to this study

**Short title:** Lipid profiles with mortality and the role of physical activity

**Correspondence to**

Der-Cherng Tarng, MD, PhD

Professor, Institutes of Physiology and Clinical Medicine, National Yang-Ming University, Taipei; and Division of Nephrology, Department of Medicine, Taipei Veterans General Hospital, Taipei, Taiwan

201, Section 2, Shih-Pai Road, Taipei 11217, Taiwan

Phone: 886-2-2871 2121 ext. 2678

Fax: 886-2-2826 4049

Email: dctarng@gmail.com

**SUPPLEMENTAL MATERIALS**

**Appendix Table 1. Mortality Risks by HDL Levels and by Physical Activity Status among Older Individuals**

**Appendix Table 2. Mortality Risks by Triglyceride Levels and by Physical Activity Status among Older Individuals**

**Appendix Table 3. Mortality Risks by Physical Activity Status among Older Individuals with Low Total Cholesterol**

**Appendix Table 4. Mortality Risks by Physical Activity Status among Individuals with Low non-HDL**

**Appendix Table 5. Mortality Risks after Classifying Baseline Physical Activity Status and Lipid Levels by Quintile.**

| **Appendix Table 1. Mortality Risks by HDL Levels and By Physical Activity Status among Older Individuals** | | | | | | | | | | | | | | |
| --- | --- | --- | --- | --- | --- | --- | --- | --- | --- | --- | --- | --- | --- | --- |
|  | **Inactive** | | | |  | **Low active** | | | |  | **High active** | | | |
| **HDL** | **Event (%)** | **Crude HR**  **(95% CI)** |  | **Adjusted HR**^*^  **(95% CI)** |  | **Event (%)** | **Crude HR**  **(95% CI)** |  | **Adjusted HR**^*^  **(95% CI)** |  | **Event (%)** | **Crude HR**  **(95% CI)** |  | **Adjusted HR**^*^  **(95% CI)** |
| **Quintile 1** | 462 (17.1) | 1 |  | 1 |  | 646 (9.7) |  |  | 1 |  | 557 (6.1) | 1 |  | 1 |
| **Quintile 2** | 240 (11.3) | 0.58 (0.50-0.68) |  | 0.70 (0.59-0.83) |  | 399 (6.7) | 0.65 (0.57-0.73) |  | 0.77 (0.68-0.88) |  | 411 (4.3) | 0.64 (0.56-0.73) |  | 0.77 (0.68-0.88) |
| **Quintile 3** | 153 (9.3) | 0.47 (0.39-0.57) |  | 0.65 (0.53-0.80) |  | 278 (5.8) | 0.55 (0.48-0.63) |  | 0.75 (0.64-0.87) |  | 307 (3.9) | 0.57 (0.49-0.65) |  | 0.76 (0.66-0.88) |
| **Quintile 4** | 230 (10.4) | 0.62 (0.53-0.73) |  | 0.82 (0.69-0.99) |  | 297 (5.0) | 0.57 (0.49-0.65) |  | 0.79 (0.68-0.92) |  | 361 (3.8) | 0.68 (0.60-0.78) |  | 0.99 (0.86-1.14) |
| **Quintile 5** | 147 (8.3) | 0.45 (0.37-0.54) |  | 0.67 (0.54-0.83) |  | 274 (5.6) | 0.57 (0.49-0.65) |  | 0.77 (0.66-0.90) |  | 305 (3.3) | 0.51 (0.45-0.59) |  | 0.73 (0.62-0.85) |
| ^*^ Adjusted for age, sex, body mass index, smoking, alcohol use, systolic blood pressure, diastolic blood pressure, hypertension, diabetes mellitus, coronary artery disease, cerebrovascular disease, white blood cell count, hemoglobin, albumin, geriatric nutritional risk index, uric acid, fasting glucose, estimated glomerular filtration rate, and urine protein level.  †Interaction for total cholesterol and physical activity status, p value=0.295.  Abbreviations: HDL, high-density lipoprotein; HR, Hazard ratio; CI, confidence interval. | | | | | | | | | | | | | | |

| **Appendix Table 2. Mortality Risks by Triglyceride Levels and By Physical Activity Status among Older Individuals** | | | | | | | | | | | | | | |
| --- | --- | --- | --- | --- | --- | --- | --- | --- | --- | --- | --- | --- | --- | --- |
|  | **Inactive** | | | |  | **Low active** | | | |  | **High active** | | | |
| **Triglyceride** | **Event (%)** | **Crude HR**  **(95% CI)** |  | **Adjusted HR**^*^  **(95% CI)** |  | **Event (%)** | **Crude HR**  **(95% CI)** |  | **Adjusted HR**^*^  **(95% CI)** |  | **Event (%)** | **Crude HR**  **(95% CI)** |  | **Adjusted HR**^*^  **(95% CI)** |
| **Quintile 1** | 294 (16.4) | 1 |  | 1 |  | 446 (8.8) |  |  | 1 |  | 489 (4.9) | 1 |  | 1 |
| **Quintile 2** | 260 (13.8) | 0.83 (0.71-0.99) |  | 1.01 (0.84-1.21) |  | 399 (7.5) | 0.84 (0.73-0.96) |  | 1.00 (0.87-1.15) |  | 424 (4.5) | 0.93 (0.81-1.06) |  | 1.06 (0.93-1.21) |
| **Quintile 3** | 240 (11.4) | 0.69 (0.58-0.81) |  | 1.03 (0.85-1.24) |  | 353 (6.2) | 0.70 (0.61-0.81) |  | 0.99 (0.86-1.16) |  | 375 (4.1) | 0.86 (0.75-0.98) |  | 1.04 (0.90-1.19) |
| **Quintile 4** | 228 (10.5) | 0.63 (0.53-0.75) |  | 1.05 (0.86-1.28) |  | 345 (5.8) | 0.65 (0.57-0.75) |  | 0.98 (0.84-1.15) |  | 331 (3.9) | 0.81 (0.71-0.93) |  | 1.05 (0.90-1.22) |
| **Quintile 5** | 210 (8.3) | 0.49 (0.41-0.59) |  | 0.88 (0.72-1.09) |  | 351 (5.7) | 0.66 (0.57-0.76) |  | 1.08 (0.92-1.27) |  | 322 (4.0) | 0.86 (0.74-0.99) |  | 1.14 (0.98-1.34) |
| ^*^ Adjusted for age, sex, body mass index, smoking, alcohol use, systolic blood pressure, diastolic blood pressure, hypertension, diabetes mellitus, coronary artery disease, cerebrovascular disease, white blood cell count, hemoglobin, albumin, geriatric nutritional risk index, uric acid, fasting glucose, estimated glomerular filtration rate, and urine protein level.  †Interaction for total cholesterol and physical activity status, p value=0.124.  Abbreviations: HR, Hazard ratio; CI, confidence interval. | | | | | | | | | | | | | | |

| **Appendix Table 3. Mortality Risks by Physical Activity Status among Older Individuals with Low Total Cholesterol ^†^** | | | |
| --- | --- | --- | --- |
| **Physical Activity Status** |  | **Hazard ratio^*^**  **(95% CI)** | **P** |
| Inactive |  | 1.29 (1.06-1.56) | 0.011 |
| Low active |  | 1.22 (1.05-1.42) | 0.011 |
| High active |  | 1.08 (0.93-1.25) | 0.308 |
| ^*^ Adjusted for age, sex, body mass index, smoking, alcohol use, systolic blood pressure, diastolic blood pressure, hypertension, diabetes mellitus, coronary artery disease, cerebrovascular disease, white blood cell count, hemoglobin, albumin, geriatric nutritional risk index, uric acid, fasting glucose, estimated glomerular filtration rate, and urine protein level.  ^†^Quintile 1 vs. Quintile 3 and Quintile 3 as reference.  Abbreviations: CI, confidence interval. | | | |

| **Appendix Table 4. Mortality Risks by Physical Activity Status among Individuals with Low non-HDL**^†^ | | | |
| --- | --- | --- | --- |
| **Physical Activity Status** |  | **Hazard ratio**^*^  **(95% CI)** | **P** |
| Inactive |  | 1.22 (1.01-1.48) | 0.038 |
| Low active |  | 1.23 (1.07-1.44) | 0.005 |
| High active |  | 1.04 (0.91-1.20) | 0.559 |
| ^*^ Adjusted for age, sex, body mass index, smoking, alcohol use, systolic blood pressure, diastolic blood pressure, hypertension, diabetes mellitus, coronary artery disease, cerebrovascular disease, white blood cell count, hemoglobin, albumin, geriatric nutritional risk index, uric acid, fasting glucose, estimated glomerular filtration rate, and urine protein level.  ^†^Quintile 1 vs. Quintile 3 and Quintile 3 as reference.  Abbreviations: CI, confidence interval. | | | |

| **Appendix Table 5. Mortality Risks after Classifying Baseline Physical Activity Status and Lipid Levels by Quintile.** | | | | | | | | | | | | |
| --- | --- | --- | --- | --- | --- | --- | --- | --- | --- | --- | --- | --- |
|  | **Inactive** | | |  | **Low active** | | |  | | **High active** | | |
|  | **Event (%)** | **Adjusted HR**^*^  **(95% CI)** | **P value** |  | **Event (%)** | **Adjusted HR**^*^  **(95% CI)** | **P value** |  | **Event (%)** | | **Adjusted HR**^*^  **(95% CI)** | **P value** |
| **Total Cholesterol** |  |  |  |  |  |  |  |  |  | |  |  |
| Quintile 1 | 486 (20.8) | 1 |  |  | 612 (10.8) | 0.66 (0.58-0.75) | <0.001 |  | 578 (6.1) | | 0.45 (0.40-0.52) | <0.001 |
| Quintile 2 | 216 (11.3) | 1 |  |  | 379 (7.0) | 0.84 (0.70-1.00) | 0.055 |  | 427 (4.7) | | 0.63 (0.53-0.76) | <0.001 |
| Quintile 3 | 205 (10.1) | 1 |  |  | 315 (5.6) | 0.71 (0.59-0.86) | <0.001 |  | 367 (4.0) | | 0.55 (0.46-0.66) | <0.001 |
| Quintile 4 | 166 (8.2) | 1 |  |  | 303 (5.3) | 0.84 (0.69-1.03) | 0.093 |  | 317 (3.5) | | 0.61 (0.49-0.74) | <0.001 |
| Quintile 5 | 159 (7.3) | 1 |  |  | 285 (5.0) | 0.96 (0.78-1.18) | 0.709 |  | 252 (3.0) | | 0.63 (0.51-0.79) | <0.001 |
| **Non-HDL** |  |  |  |  |  |  |  |  |  | |  |  |
| Quintile 1 | 424 (19.0) | 1 |  |  | 541 (9.9) | 0.68 (0.59-0.78) | <0.001 |  | 555 (5.8) | | 0.47 (0.41-0.54) | <0.001 |
| Quintile 2 | 244 (12.1) | 1 |  |  | 406 (7.3) | 0.78 (0.66-0.93) | 0.005 |  | 430 (4.5) | | 0.55 (0.47-0.66) | <0.001 |
| Quintile 3 | 201 (10.4) | 1 |  |  | 307 (5.5) | 0.68 (0.57-0.83) | <0.001 |  | 363 (4.0) | | 0.57 (0.47-0.68) | <0.001 |
| Quintile 4 | 192 (9.8) | 1 |  |  | 310 (5.7) | 0.78 (0.64-0.94) | 0.010 |  | 289 (3.4) | | 0.52 (0.43-0.63) | <0.001 |
| Quintile 5 | 171 (7.3) | 1 |  |  | 330 (5.4) | 0.99 (0.81-1.21) | 0.940 |  | 304 (3.6) | | 0.73 (0.60-0.90) | 0.003 |
| **HDL** |  |  |  |  |  |  |  |  |  | |  |  |
| Quintile 1 | 462 (17.1) | 1 |  |  | 646 (9.7) | 0.71 (0.62-0.81) | <0.001 |  | 557 (6.1) | | 0.53 (0.46-0.61) | <0.001 |
| Quintile 2 | 240 (11.3) | 1 |  |  | 399 (6.7) | 0.77 (0.65-0.92) | 0.003 |  | 411 (4.3) | | 0.56 (0.47-0.67) | <0.001 |
| Quintile 3 | 153 (9.3) | 1 |  |  | 278 (5.8) | 0.85 (0.69-1.05) | 0.136 |  | 307 (3.9) | | 0.63 (0.51-0.78) | <0.001 |
| Quintile 4 | 230 (10.4) | 1 |  |  | 297 (5.0) | 0.71 (0.59-0.85) | <0.001 |  | 361 (3.8) | | 0.59 (0.49-0.71) | <0.001 |
| Quintile 5 | 147 (8.3) | 1 |  |  | 274 (5.6) | 0.86 (0.70-1.06) | 0.165 |  | 305 (3.3) | | 0.53 (0.43-0.65) | <0.001 |
| ^*^ Adjusted for age, sex, body mass index, smoking, alcohol use, systolic blood pressure, diastolic blood pressure, hypertension, diabetes mellitus, coronary artery disease, cerebrovascular disease, white blood cell count, hemoglobin, albumin, geriatric nutritional risk index, uric acid, fasting glucose, estimated glomerular filtration rate, and urine protein level.  Abbreviations: HDL, high-density lipoprotein; HR, Hazard ratio; CI, confidence interval. | | | | | | | | | | | | |
